# Koopman-Based Model Predictive Control of Functional Electrical Stimulation for Ankle Dorsiflexion and Plantarflexion Assistance

**DOI:** 10.1109/TNSRE.2025.3551933

**Published:** 2025-04-04

**Authors:** Mayank Singh, Noor Hakam, Trisha M. Kesar, Nitin Sharma

**Affiliations:** Department of Electrical Engineering, North Carolina State University, Raleigh, NC 27606 USA; UNC/NC State Lampe Joint Department of Biomedical Engineering, NC State University, Raleigh, NC 27606 USA; Division of Physical Therapy, Department of Rehabilitation Medicine, Emory University School of Medicine, Atlanta, GA 30322 USA.; UNC/NC State Lampe Joint Department of Biomedical Engineering, NC State University, Raleigh, NC 27606 USA

**Keywords:** Functional electrical stimulation (FES), extended dynamic mode decomposition (EDMD), model predictive control (MPC), gait assistance, nonlinear dynamics

## Abstract

Functional Electrical Stimulation (FES) can be an effective tool to augment paretic muscle function and restore normal ankle function. Our approach incorporates a real-time, data-driven Model Predictive Control (MPC) scheme built upon a Koopman operator theory (KOT) framework. This framework adeptly captures the complex nonlinear dynamics of ankle motion in a linearized form, enabling the application of linear control approaches for highly nonlinear FES-actuated dynamics. Our method accurately predicts the FES-induced ankle movements, accounting for nonlinear muscle actuation dynamics, including the muscle activation for both plantarflexors and dorsiflexors (Tibialis Anterior (TA)). The linear prediction model derived through KOT allowed the formulation of the MPC problem with linear state space dynamics, enhancing the FES-driven control’s real-time feasibility, precision, and adaptability. We demonstrate the effectiveness and applicability of our approach through comprehensive simulations and experimental trials, including three participants with no disability and a participant with Multiple Sclerosis. Our findings highlight the potential of a KOT-based MPC approach for FES-based gait assistance that offers effective and personalized assistance for individuals with gait impairment conditions.

## I. Introduction

NEUROLOGICAL conditions such as stroke, spinal cord injury (SCI), cerebral palsy, and multiple sclerosis (MS) often impair ankle function, necessitating specialized rehabilitation interventions. Functional Electrical Stimulation (FES) can restore ankle function by eliciting artificial muscle contractions in paralyzed plantarflexor and dorsiflexor muscles through the application of noninvasive electrical stimulation, thereby facilitating improved joint function [1].

To effectuate a natural and efficient walking pattern, FES-based ankle assistance requires accurate timing and modulation of the stimulation input to the Gastrocnemius (GAS) muscle for plantarflexion during the push-off phase and to Tibialis Anterior (TA) for dorsiflexion during the swing phase of the gait cycle. Various control strategies for ankle rehabilitation [2], [3], [4] demonstrate the effectiveness of FES in gait rehabilitation. Gil-Castillo et al. [5] comprehensively review FES based control methods for assisting ankle function.

Among contemporary control methodologies, Iterative Learning Control (ILC) has been often used for FES-based assistance to correct ankle condition, called drop-foot [6]. ILC schemes are often model-free or rely on linear time-invariant dynamics, simplifying implementation. For instance, Seel et al. [6] used ILC with six inertial sensors to estimate ankle angles in post-stroke patients, achieving rapid convergence but introducing discontinuities by resetting control inputs after each gait cycle and requiring substantial sensor data. Page and Freeman [7] improved the ILC design by developing a continuous repetitive control scheme, eliminating reinitialization, reducing computational burden, and enhancing trajectory tracking. Similarly, Jiang et. al. [8] designed a framework for dorsiflexion assistance using dual parameters to reduce stimulation intensity and mitigate muscle fatigue. Müller et. al. [9] extended ILC to assist both knee and ankle motion, showing adaptability for individual stimulation patterns but requiring refinement to address sensitivity to knee angle resets.

ILC-based FES designs, including those in [7] and [8], are often developed under linear system assumptions without explicitly addressing nonlinearities in muscle recruitment. While these approaches have demonstrated impressive kinematic tracking for cyclical tasks of walking, their reliance on linearized models leave room for exploring nonlinear approaches, which can lead to better stimulation design, mitigating its adverse effects. Nonlinear control of FES for ankle control was demonstrated by Zhang et. al. [10], where ultrasound-derived muscle activation was integrated into a nonlinear model for drop foot correction. However, their state feedback-based dynamic surface controller (DSC) lacked optimization of a performance index and constraint handling of FES input constraints, which may risk overstimulation and rapid muscle fatigue.

Moreover, results on FES based gait assistance/improvement presented in [6], [7], [8], [9], and [10] have primarily focused on drop foot correction. In these results, FES mainly targets the TA muscle during the swing phase, foregoing stimulation of plantarflexors, which are critical for push-off [11]. Efficacy of FES stimuli to plantarflexor muscles has been shown for correcting post-stroke gait deficits [3] and improving walking after SCI [12]. It was noted in [3] and [11], that applying stimulation to both plantarflexors and dorsiflexors results in improved gait that is closer to the normal gait cycle in chronic stroke survivors. Despite the evidence on the significance of FES-elicited plantarflexion, closed-loop control of both FES-evoked plantarflexion and dorsiflexion remains unexplored.

In this paper, we present a novel Koopman Model Predictive Control (KMPC) framework for Functional Electrical Stimulation (FES)-based gait assistance, applying optimally designed stimulation signals to both plantarflexor and dorsiflexor muscles throughout the gait cycle. By employing Koopman Operator Theory (KOT), we capture the system’s nonlinearities through the linear evolution of lifted observable functions of the states, facilitating the application of linear control techniques for the MPC framework. We derive a linear representation of the inherently nonlinear ankle dynamics, enabling real-time prediction and control of the full gait cycle. The data-driven operator converts the nonlinear ankle motion dynamics into linear dynamics, which eases MPC formulation and real-time implementation [13], [14], [15]. The KMPC formulation performs optimal feedback control in real-time, utilizing the Koopman-based FES actuated ankle model to solve the moving horizon optimization problem under FES stimulation input constraints.

For ankle assistance control, [16] used an MPC to design optimal muscle excitation for the TA muscle for adequate toe/foot clearance. While constraints on ankle and control inputs were considered, a formal closed-loop stability and control feasibility analysis were missing. As the effectiveness of MPC depends on model accuracy, necessitating extensive system identification, especially for complex neuromuscular dynamics. Addressing nonlinear dynamics and constraints can increase computational demands, posing challenges for realtime implementation. To mitigate these issues, [17] used a Koopman-based data-driven MPC control to calculate optimal FES stimulation for the TA muscle to correct drop foot during the swing phase to avoid toe drag. In this paper, we extend the data-driven MPC to design optimal FES stimulation for ankle assistance for both plantarflexors and dorsiflexors to provide assistance during a complete gait cycle. To the best of our knowledge, this is the first implementation of an FES-based optimal control strategy for the gait cycle in real-time.

The paper is organized as follows – II describes the ankle dorsiflexion and plantarflexion motion dynamics actuated under FES. III discusses the overview of the Koopman-based data-driven model of ankle dynamics and the subsequent formulation of the MPC-based control synthesis problem in Section IV. Experimental setup, simulation, and experiment results are presented in V. VI includes a discussion on experimental results and their comparison with existing FES-based ankle assistance approaches, limitations of the KMPC approach, and future directions.

## II. Ankle Joint Gait dynamics

During a gait cycle, the ankle movement is modeled as continuous dynamics within swing and stance phases with a discrete transition event between the two phases. Therefore, the ankle dynamics, modeled as a switched system to accommodate for the transition, is given as

(1) [JPθ¨+fJP(θ,θ.)JDθ¨+fJD(θ,θ.)]={τP+τextt∈tstτD,t∈tsw}

where the net torque about the ankle is defined as $\tau^{\zeta }=g_{J}^{\zeta }(\theta ,\dot{\theta }u^{\zeta })\in R$, where ζ=P, D represent the ankle dynamics driven by ankle plantarflexors during the stance phase and ankle dorsiflexors during the swing phase, respectively. The net torque terms, $g_{J}^{\zeta }(\theta ,\dot{\theta }u^{\zeta })$, include the torque-angle and torque-angular velocity terms, and and $u^{\zeta }\in R$, which is the FES modulated parameter (current, pulse width, or frequency) applied on the GAS, and TA muscles [18]. During the stance phase, the ankle torque is influenced not only by muscle activations (modulated by $u^{P}$) but also by external torque, $\tau_{ext}=r(\theta )F_{GRF}(t)$, due to an additional moment arising from the ground reaction force (GRF) $F_{GRF}(t)$ acting with a moment arm, r(θ), from the ankle joint to the point of application of the GRF.

The stance and swing phases are timed as $t_{st}\;≔[t_{start},t_{stance}]$ and $t_{sw}≔[t_{swing},t_{end}]$, respectively. $J^{\zeta }\in R^{+}$ is the unknown inertia term of the foot along the dorsiflexion and plantarflexion axis of rotation, and θ(t), $\dot{\theta }(t)$, and $\ddot{\theta }(t)\in R$ denote the angular position, angular velocity, and angular acceleration, respectively. $f_{J}^{\zeta }(\theta ,\dot{\theta })$ in (1) is composed of the musculoskeletal viscosity torque term, musculoskeletal elasticity, and the gravitational term. The explicit definitions of the functions can be obtained from [18].

For each phase $t_{st}$ and $t_{sw}$, we can rewrite the system dynamics in (1), by selecting $\theta_{1}\;=\;\theta$ and $\theta_{2}\;=\;\dot{\theta }$. The equivalent state space representation for can be formulated as

(2) x.a={faP(xa)+gaP(xa,uP,t)∀t∈tstfaD(xa)+gaD(xa,uD)∀t∈tsw}

where ${\dot{x}}_{a}={\left[{\dot{\theta }}_{1}\;{\dot{\theta }}_{2}\right]}^{T}$, $f_{a}^{\zeta }(x_{a})\in R^{2}$ are the system dynamics, and $g_{a}^{\zeta }(x_{a},u^{\zeta })\in R^{2}$ are the actuation dynamics.

We can now set up the optimal tracking problem by defining a tracking error $e(t)\in R^{2}$, which is defined as

(3) $$e=x_{a}-x_{d},$$

where $x_{d}\in R^{2}$ is a bounded desired trajectory for the desired position and velocity. It is assumed that $x_{d}$ and its first derivative, ${\dot{x}}_{d}=h_{d}(x_{d})\in R^{2}$, are *Lipschitz* continuous.

By defining an augmented state as $x={\left[e^{T}\;x_{d}^{T}\right]}^{T}\in R^{3}$, the system dynamics can be written as $\dot{x}=f^{\zeta }(x)+g^{\zeta }(x,u^{\zeta })$, where the system matrices $f^{\zeta }(x)$ and $g^{\zeta }(x,u^{\zeta })$ matrices become $f^{\zeta }(x)=\left[\begin{array}{c} f^{\zeta }(e+x_{d})-h_{d}(x_{d})\\ h_{d}(x_{d}) \end{array}\right]$; $g^{\zeta }(x,u^{\zeta })=\left[\begin{array}{c} g^{\zeta }(e+x_{d})\\ 0 \end{array}\right]$.

Using zero order hold approximation the continuous-time system above can be discretized and described as

(4) xk+1={fP(xk)+gP(xk,ukP)∀t∈tstfD(xk)+gD(xk,ukD)∀t∈tsw}

We can define an indicator function $\sigma_{k}$ based on the gait phase time intervals for the stance and swing phases as

(5) σk={0∀t∈tst1∀t∈tsw,}

where the phase indicator $\sigma_{k}$ takes the value 0 for the stance phase and 1 for the swing phase. Upon incorporating the phase indicator the complete ankle motion dynamics during a gait cycle can then be described as

(6) $$x_{k+1}=(1-\sigma_{k})\left(f^{P}(x_{k})+g^{P}(x_{k},u_{k}^{P})\right)\;\;+\sigma_{k}\left(f^{D}(x_{k})+g^{D}(x_{k},u_{k}^{D})\right).$$

*Assumption 1:* Based on human ankle kinematic data [19], ankle position, velocity, and moment are continuous. Therefore, at slow gait cycle speed, $f^{\zeta }(.)$ and $g^{\zeta }(.)$ are assumed to be Lipschitz at the switching instant. We utilize this assumption in subsequent sections to derive the linear predictor model using Koopman operator. Switching criteria for similar systems with continuous states, but discrete actuation have been considered in [20], where the switching between different muscle groups is represented by their respective actuation matrices which are bounded.

## III. Koopman-Based Model Predictive Control

This section provides the mathematical framework for predicting the nonlinear ankle joint dynamics actuated by FES using Koopman Operator Theory (KOT).

### A. Prediction/Identification

We consider the dynamics in 4 where the controlled state $x_{k}\in 𝒳$, input $u_{k}\in 𝒰$ are sampled to form a finite set, ${𝒩}_{c}$. The Koopman operator acts on a function space ℱ of mapping from 𝒳 into R, referred to as observables. The Koopman operator, 𝒦, is an infinite-dimensional linear operator that models the time-based evolution of a composite function $\Lambda (x_{k})\in R^{\infty }$, which act as the *koopman observables*, forward in time. Koopman operators are parameterized by $x_{k}$, $u_{k}$ as follows

(7) $$𝒦\Lambda (x_{k})=\Lambda (f(x_{k},u_{k})),\;\;\;\Lambda \in ℱ$$

where 𝒦 maps observables to the original state space dynamics in 4. A subspace is Koopman-invariant if

(8) $$𝒦\Lambda \in \bar{ℱ},\;\;\forall \Lambda \in \bar{ℱ},\;\forall u\in 𝒰$$

A dictionary of observables, $\chi \;:𝒳\to R^{𝒩}$, is Koopman-invariant if its elements span a Koopman-invariant subspace. Since evaluation of the dictionary often involves “lifting” of the original state vector to a higher dimensional space, $\chi (x_{k})$, which is commonly referred to as the lifted state. $\chi (x_{k})$ is composed of the original state themselves, or nonlinear functions of state that are *Lipschitz* continuous. The extension to non-autonomous systems has been researched extensively recently, see [13], [14], [21]. The extension to non-autonomous system is given as

(9) $$𝒦(\Lambda (x_{k},u_{k}))=\Lambda (f(x_{k},u_{k}),\;h(x_{k},u_{k}))\;\forall \Lambda \in ℱ,$$

While this operator renders an infinite-dimensional system and accurately describes a nonlinear system through a linear system, but is practically infeasible to implement. For practical feasibility, the infinite-dimensional operator, 𝒦, is approximated using a finite dimensional operator, defined as $\tilde{𝒦}$, which is calculated using the Extended Dynamic Mode Decomposition (EDMD) [13].

To derive the Koopman operators for each phase, we collect the time-series data snapshots of the state data as ${\left\{x_{k}\right\}}_{k=1}^{M}$ where $x_{k}$ represents the state at time step k, and control input data as ${\left\{u_{k}^{\zeta }\right\}}_{k=1}^{M}$ where $u_{k}^{\zeta }$ represents the control input at time step k during the stance and swing phases.

We define the lifted-space Koopman observable, $\Psi_{k}(x,u)\in R^{P}$, to set up an EDMD problem to predict the linear evolution of the Koopman observable vector using

(10) $$\Psi_{k+1}(x,u)=\tilde{𝒦}\Psi_{k}(x,u),$$

where $\tilde{𝒦}$ is the finite-dimensional Koopman operator which maps the lifted-state observables forward in time. Using the state and control time-series snapshots we populate the lifted-space matrices, as

$$\;{{𝒟}^{\zeta }}_{k}=\left[\Psi (x_{1},u_{1}^{\zeta })\cdots \Psi (x_{M-1},u_{M-1}^{\zeta })\right]\;{𝒟}_{k+1}^{\zeta }=\left[\Psi (x_{2},u_{2}^{\zeta })\cdots \Psi (x_{M},u_{M})\right]$$

where ${{𝒟}^{\zeta }}_{k}$, ${{𝒟}^{\zeta }}_{k+1}\in R^{P\times M}\;\forall \;k=1,\ldots ,M$, are the collected observable block snapshots from FES inputs and IMU state measurements for each gait phase. The Koopman observable vector dynamically evolves as

(11) $${𝒟}_{k+1}=\tilde{𝒦}{𝒟}_{k},$$

where ${𝒟}_{k}\;=\;\Psi_{k}(x,u)={\left[\Psi_{x}(x_{k})\;\Psi_{u}(u_{k})\right]}^{T}$. To obtain the control state and control flow maps in the lifted space, the approximated Koopman operator, $\tilde{𝒦}$ can be further subdivided as

(12) $$\tilde{𝒦}=\left[\begin{array}{cc} {\tilde{𝒦}}_{xx} & {\tilde{𝒦}}_{xu}\\ {\tilde{𝒦}}_{ux} & {\tilde{𝒦}}_{uu} \end{array}\right],$$

where ${\tilde{𝒦}}_{xx}$ represents the influence of the state observables, $\Psi_{x}(x_{k})$, on the future state observables, and ${\tilde{𝒦}}_{xu}$ represents the influence of the control observables, $\Psi_{u}(u_{k})$, on the future state observables. The terms ${\tilde{𝒦}}_{ux}$, ${\tilde{𝒦}}_{uu}$ in 12 refers to mappings that evolve the observations on control which are ignored here.

To determine the Koopman operator for each phase, ζ={P−stancephase,D−swingphase}, we set up a least-squares regression problem wherein the error difference between the observed next step data ${𝒟}_{k+1}$, and the prediction from ${\tilde{𝒦}}^{\zeta }{𝒟}_{k}$, described as

𝒦~ζ=argmin𝒦~∑k=0M−1‖[Ψx(xk+1)Ψu(uk+1)]‖−‖[𝒦~xxζ𝒦~xuζ𝒦~uxζ𝒦~uuζ][Ψx(xk)Ψu(uk)]‖2

The least-squares solution for $\tilde{𝒦}$ is given as

(13) $$\tilde{𝒦}=FG^{†},$$

where

(14) $$F=\frac{1}{M}\sum_{k=0}^{M-1}{𝒟}_{k+1}{𝒟}_{k}^{T},\;G=\frac{1}{M}\sum_{k=0}^{M-1}{𝒟}_{k}{𝒟}_{k}^{T},$$

where pseudoinverse $G^{†}$ is utilized. Using the indicator function in 5 and the phase-based Koopman operator, the ankle motion dynamics during a complete gait cycle can then be represented as

(15) $$[\Psi_{x}(x_{k+1})]=(1-\sigma_{k})\left[{\tilde{𝒦}}_{xx}^{P}\;{\tilde{𝒦}}_{xu}^{P}\right]\left[\Psi_{k}(x,u^{P})\right]\;\;+\sigma_{k}\left[{\tilde{𝒦}}_{xx}^{D}\;{\tilde{𝒦}}_{xu}^{D}\right]\left[\Psi_{k}(x,u^{D})\right]$$

To obtain the prediction dynamics for the original state in (4), we compute the flow map between lifted-space observables, $\Psi_{k}(x,u)$ and original state dynamics, $x_{k}$. We redefine the state vector $x_{k}$ as $z_{k}$ to avoid any notational confusion with (II). To recover $z_{k}$, we can describe the mapping between Koopman observable, $\Psi_{k}(x,u)$, and $z_{k}$ as $z_{k}\;=\;C\Psi_{k}(x,u)$, where $C\in R^{3\times P}$ denotes the mapping. To obtain C, we solve the following least-squares problem

(16) $$\text{arg}\;\underset{C}{\text{min}}\;\sum_{k=0}^{M-1}\frac{1}{2}{\left\|C\Psi_{k}(x,u)-z_{k}\right\|}^{2}.$$

By solving (16), and plugging $\Psi_{k}(x,u)=C^{-1}z_{k}$ into the lifted-space flow map 10, we obtain the linear prediction model for phase-based FES-driven ankle motion dynamics during a complete gait cycle as

(17) $$z_{k+1}={\tilde{A}}^{\zeta }z_{k}+{\tilde{B}}^{\zeta }u_{k}^{\zeta },$$

where

(18) $${\tilde{A}}^{\zeta }=\tilde{C}{\tilde{𝒦}}_{xx}^{\zeta }C^{-1};\;{\tilde{B}}^{\zeta }=C{\tilde{𝒦}}_{xu}^{\zeta }C^{-1}.$$

Using the indicator function, $\sigma_{k}$, the combined state dynamics can be written as

(19) $$z_{k+1}=(1-\sigma_{k})\left({\tilde{A}}^{P}z_{k}+{\tilde{B}}^{P}u_{k}^{P}\right)\;\;+\sigma_{k}\left({\tilde{A}}^{D}z_{k}+{\tilde{B}}^{D}u_{k}^{D}\right)$$

where $z_{k}={\left[e^{T}\;x_{d}^{T}\right]}^{T}\in R^{3}$ is the state vector. $A^{P}$, $A^{D}\in R^{3\times 3}$, $B_{1}$, $B_{2}\in R^{3\times 1}$ are the Koopman operator based linear state space mappings, and $u_{P}^{k}$, $u_{k}^{D}\in R$ are the FES control input vector for assisting ankle plantarflexion and dorsiflexion during a gait cycle.

### B. Koopman Observables

The choice of basis functions for constructing the dictionary of observables significantly impacts the performance of the Koopman operator [13]. Appropriate choice for basis functions can be found in [22]. The accuracy of the Koopman operator improves with the length, P, of the observable vector, $\Psi_{k}(x,u)$. As P→∞, the Koopman operator, $\tilde{𝒦}$, accurately describes linear prediction dynamics for the original nonlinear system [23].

We set a prediction accuracy threshold, ${\left\|x_{k}-z_{k}\right\|}^{2}\leq \eta$, for η≤0.5 RMSE for ankle motion during a gait cycle. We achieved the threshold for P=13. For the ankle assistance control with state as joint angles -θ(t) and $\dot{\theta }(t)$, our Koopman observable library included a custom library: linear terms $\text{-}\;\theta_{1}$, $\theta_{2}$, ${\dot{\theta }}_{1}$, ${\dot{\theta }}_{2}$, and nonlinear terms $\text{-}\;sin(\theta_{1})$, $cos(\theta_{1})$, $sin(\theta_{2})$, $cos(\theta_{2})$, $\theta_{1}^{2}$, $\theta_{2}^{2}$, $\theta_{1}\theta_{2}$, ${\dot{\theta }}_{1}{\dot{\theta }}_{2}$. The choice of observable is dictated by the ankle dynamics that exhibit nonlinear effects due to muscle activation, phase transitions, and joint stiffness. Observables like sin(θ) and $\theta^{2}$ can theoretically capture such effects by approximating periodic and quadratic relationships seen in ankle motion, respectively. Including higher-order terms (e.g., ${\dot{\theta }}^{2}$, sin(θ)) helps approximate the nonlinearities associated with force-length and force-velocity relationships in muscle dynamics.

#### Remark 1 (Koopman Invariance):

To maintain Koopman invariance, the observables should be chosen to cover the system’s entire dynamic range, while upholding Assumption 1. Theoretically, the observables should be designed to provide a stable, controllable Koopman linear system approximation. Also, to uphold Assumption 1, we use the same set of observables for both stance and swing phase.

### C. Koopman Model Prediction Accuracy

**Fig. 1.**
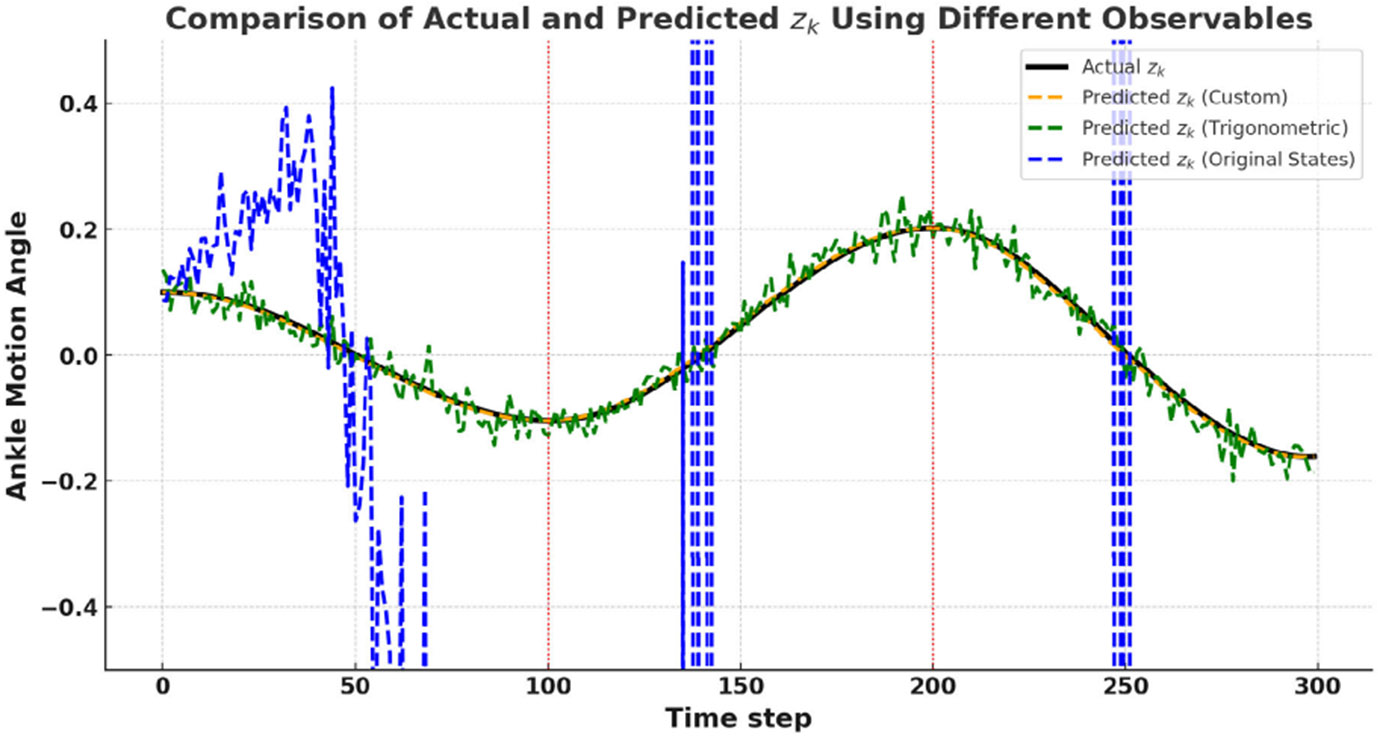
*Prediction results* - Plot shows the ankle motion prediction during a gait cycle under test FES actuation for different observables (*states*, *custom*, *trigonometric*). The dynamics approximated from (19) are utilized to predict the approximate dynamics.

Accuracy of 𝒦 is tested with simulation results. Simulation were performed by using the parameters from [18] with different initial conditions to obtain the samples of actual system trajectories. The dataset used to train the Koopman-based MPC framework for gait rehabilitation was derived from 150 gait cycles. Each gait cycle consists of 200 samples, with an even split between the stance and swing phases to capture phase-specific dynamics. This sampling approach resulted in a dataset with approximately 30, 000 samples. To construct the dataset, we first sample initial states $\left(x,\dot{x},u_{fes})\in [25,-20]\times [-2,2]\times [0,50\right]$. Here, x represents the initial position, $\dot{x}$ represents the initial velocity, and $u_{fes}$ denotes the FES (Functional Electrical Stimulation) input level. These ranges were chosen to account for variability in patient gait patterns, the extent of ankle joint movement during gait cycles. The control inputs were linearly varied within the range of 0 to 30mA. This ensured that the dataset captured the system’s response to different stimulation levels. A sampling frequency of 200 Hz was used. With the generated dataset the Koopman operator was designed. Based on the prediction dynamics, simulation results for a nominal sinusoidal trajectory tracking of the ankle joint dynamics for different observables are given in Fig. (1).

**Fig. 2.**
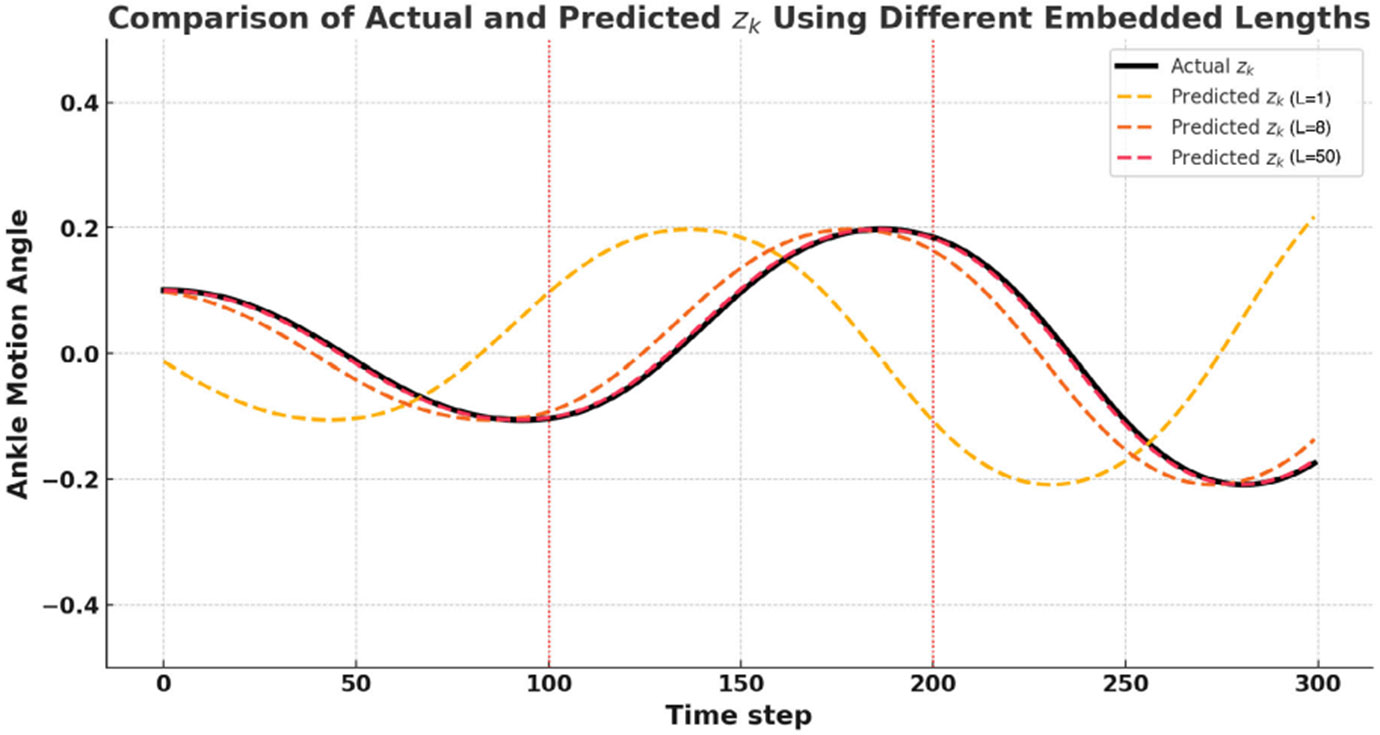
Comparison of actual and predicted ($z_{k}$) ankle motion angles using different embedding lengths (L) in the Koopman-based prediction framework. The black solid line represents the actual ankle motion trajectory, while the dashed lines correspond to predictions with embedding lengths L=1 (yellow), L=8 (orange), and L=50 (red). Increasing the embedding length improves prediction accuracy, as evidenced by the closer alignment of the L = 50 prediction with the actual trajectory. The results highlight the importance of appropriate embedding length selection in achieving accurate Koopman-based predictions.

Another important criteria for prediction accuracy of ${\tilde{𝒦}}^{\zeta }$ is the number of past states considered in the observables referred as the embedding length. We considered different sample ranges to compare the prediction accuracy of the approximated Koopman operator, ${\tilde{𝒦}}^{\zeta }$, for different embedding lengths. Prediction for sample ranges L=1, 8, 50 are plotted in Fig. (2).

## IV. Data-Driven Model Predictive control

### A. Koopman Model Predictive Control

Let the decision and state variables be defined as

(20) $$z_{k}=[z_{k|k}^{i}\cdots z_{k+N|k}^{i}];$$

(21) $$u_{k}=[u_{k|k}^{i}\cdots u_{k+N-1|k}^{i}],$$

where the vectors $z_{k}$, $u_{k}\in R^{3}$, R are the state and control vectors written in the standard MPC notation.. Using the indicator function in (5), we can describe both the stance and swing phase, timed as [$T_{start}$, $t_{stance}$] and [$t_{swing}$, $T_{end}$], linear prediction dynamics. The model predictive problem can then be formulated as follows

(22) $$\underset{u^{p},u^{d}}{\text{min}}\;\;J(z_{k},u_{k|k})=\sum_{i=1}^{T_{U}}l(.)+\;V_{T_{N}}$$

subject to $z_{k+1+j|k}=(1-\sigma_{k})\left(A^{P}z_{k}+B^{P}u_{k}^{P}\right)$

(23) $$+\sigma_{k}\left(A^{D}z_{k}+B^{D}u_{k}^{D}\right)\;(a)\;z_{k|k}\in \Omega_{\chi }^{\zeta },\;u_{k|k}\in \Omega_{\upsilon }^{\zeta }\;(b)\;\Delta z_{k+T_{N}}\in \Omega_{\chi^{+}},\;(c)$$

where $l(.)={\left\|{\bar{z}}_{k+1}^{T}\right\|}_{Q_{1}}^{2}+(1-\sigma_{k}){\left\|u_{k+1}^{P^{T}}\right\|}_{R^{\zeta }}^{2}+\sigma_{k}{\left\|u_{k+1}^{D^{T}}\right\|}_{R^{\zeta }}^{2}$ and $V_{T_{N}}=z_{k+T_{U}}^{T}S^{\zeta }z_{k+T_{U}}$ are the running and terminal cost. $T_{U}$ is the prediction horizon. Based on the indicator function, $\sigma_{k}$, $T_{U}$ represents the prediction horizon for the gait intervals $t_{st}$ and $t_{sw}$. The indicator function during experiments is implemented based on ground reaction forces (GRF) which is non-zero during the stance phase and zero during the swing phase. The running cost, l(.), is the performance measure penalizing the kinematic state and control inputs considered over the control horizon, $T_{U}$, for both stance and swing phase. $Q\;\in \;R^{2\times 2}$ and R∈R are *positive definite* weighting matrices penalizing the individual states and control inputs and ensures l and V are positive definite (PD) and radially unbounded (RU). $S^{\zeta }\in R^{2\times 2}$ is the terminal cost weighting matrix. $\Omega_{\upsilon }$ denotes the FES stimulation bounds and $\Omega_{\chi }$ denotes the set of the state constraints. (As the current time step is fixed based on the number of samples, $z_{k}$ will be used instead of $z_{k|k}$, and system matrices derived over M samples will be denoted by $A^{\zeta }$, $B^{\zeta }$ to simplify the notations). $\Omega_{\chi^{+}}$ denotes the terminal set defined to ensure that the state remains within a stabilizable region at the end of the prediction horizon. Gait phase based terminal weighting matrix

(24) Sζ={SP,ifσk=0SD,ifσk=1,}

is derived for both the stance and swing phase by solving the discrete-time algebraic Riccati equation (DARE)

(25) $$S^{\zeta }={\tilde{𝒦}}_{\;xx}^{T^{\zeta }}S^{\zeta }{\tilde{𝒦}}_{xx}^{\zeta }\;\;-{\tilde{𝒦}}_{\;xx}^{T^{\zeta }}S^{\zeta }{\tilde{𝒦}}_{xu}^{\zeta }{\left(R+{\tilde{𝒦}}_{\;xu}^{T^{\zeta }}S^{\zeta }{\tilde{𝒦}}_{xu}^{\zeta }\right)}^{-1}{\tilde{𝒦}}_{\;xu}^{T^{\zeta }}S^{\zeta }{\tilde{𝒦}}_{x}^{\zeta }\;\;+Q,$$

where ${\tilde{𝒦}}_{xx}^{\zeta }$, ${\tilde{𝒦}}_{xu}^{\zeta }$ are obtained from the Koopman prediction model.. We define the terminal set as $\Omega_{\chi^{+}}=\{z\;|\;{\left(z_{k+T_{U}}\right)}^{T}S^{\zeta }z_{k+T_{U}}\leq \epsilon \}$, such that there exists a stabilizing terminal control law, such as an LQR policy, where $u_{T_{N}}^{\zeta }=\pi (z_{T_{N-1}})\in \Omega_{\upsilon }^{\zeta }$ which ensures that the closed-loop stability criteria, $V_{T_{N}+1}\leq V_{T_{N}}-(l({\bar{z}}_{T_{N}},{\bar{\tau }}_{T_{N}-1},{\bar{\phi }}_{T_{N}-1}))$, is satisfied for the MPCA problem in 22. The set is parameterized by ϵ which ensures that the terminal state remains bounded and controllable.

## V. Experimental Results

### A. Data Collection

The study was approved by the Institutional Review Board (at North Carolina State University (IRB Protocol number: 20602).

#### Participants:

Three non-disabled subjects (A1, A2, A3, age: 27.4 ± 3.1 years, height: 1.73 ± 0.15 m, mass: 82.0 ± 7.1 kg) without any neuromuscular or orthopedic disorders were recruited. One subject (S1, age: 62years, height 1.53 m, mass: 49kg) with multiple sclerosis (MS) was recruited.

#### System ID task:

For the data collection pertaining to walking tasks, the experimental setup was designed to capture the dynamics of gait on a treadmill. Both non-disabled and MS subjects, designated as A1, A2, and A3, and S1, respectively, walked at a controlled speed of 0.1m∕s, 0.2m∕s, and 0.3m∕s, to accommodate the slow speed and no volition of subject with Multiple Sclerosis (MS). This setup aimed to collect comprehensive Inertial Measurement Unit (IMU) data reflecting joint angles, as well as stimulation currents (plantarflexion = 10−25mA, dorsiflexion = 10−20mA, frequency = 33Hz) directed at the TA and GAS muscles. The stimulation parameters, specifically the current and frequency, were maintained consistently across trials, with FES stimulation current as decision variable. This approach allowed for the collection of detailed data on how varying the control input influence the muscles’ response during the walking task for accurate Koopman operator derivation. Each subject underwent three trials for the first two sessions to ensure a robust data set for accurate koopman operator derivation.

#### Experimental protocol:

A wearable sensing system, based on [24], was used to measure the ankle joint kinematics. Along with measuring the ankle kinematics, IMU and ground reaction forces (GRF) measurements were also used for gait phase detection based on methods discussed in [25],. A realtime target machine (Speedgoat Inc., Liebefeld, Switzerland) was used for experiments with integrated GRF, IMU signals, and FES stimulation through MATLAB 2019b. The data is sampled at a sampling frequency of 200 Hz. The prediction horizon, $T_{U}$, is selected based on the average duration of a gait cycle of individuals, which was an average of 2–4 seconds for the speeds 0.1m∕s, 0.2m∕s, and 0.3m∕s. A prediction horizon of 100–200 ms were chosen.

To prevent muscle fatigue, particularly in the TA and GAS muscle, sufficient rest periods were integrated into the experimental protocol. The treadmill was equipped with GRF sensors. GRF measurements enabled describing the indicator function, $\sigma_{k}$ which facilitates precise triggering of the stance and swing phases’ FES stimulation. The data garnered from these walking tasks, including IMU readings of joint angles and FES stimulation details, were used to populate the observable matrix.

### B. Experiments & Results

**Fig. 3.**
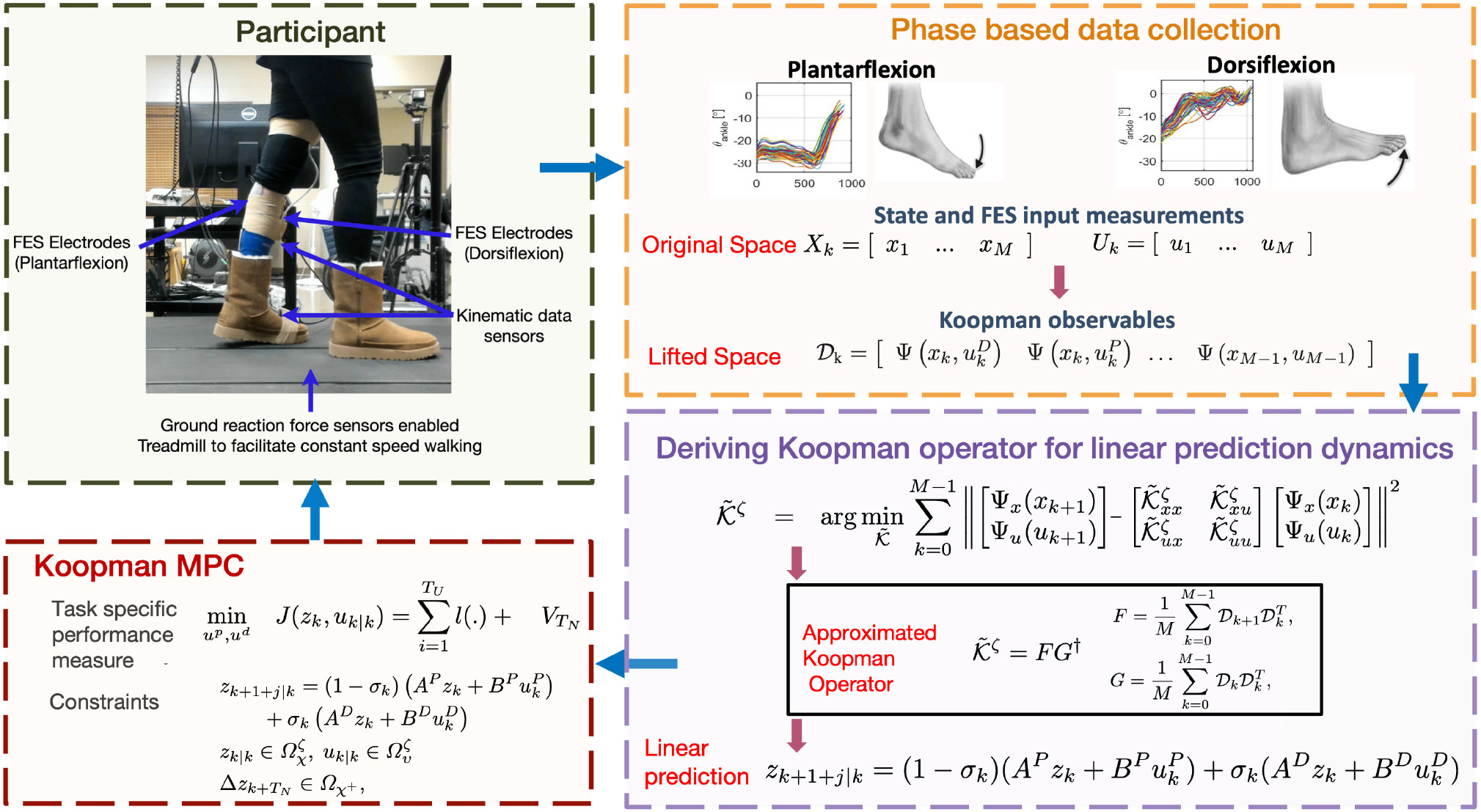
The experimental setup DDMPC framework for FES-driven gait assistance are illustrated - . The participant walks on a treadmill equipped with ground reaction force (GRF) sensors to detect gait phase transitions (stance and swing phases). FES electrodes are placed on the Gastrocnemius (GAS) and Tibialis Anterior (TA) muscles to induce plantarflexion and dorsiflexion, respectively, with stimulation parameters set at f=33Hz, $i=u_{k|k}\;\mathrm{mA}$. Kinematic data sensors record ankle motion dynamics, while the treadmill enables constant-speed walking. Phasebased data collection captures state measurements ($x_{k}$) and FES inputs ($u_{k}$) during walking, dividing the gait cycle into stance and swing phases. The raw data is lifted to a higher-dimensional space using Koopman observables, which capture nonlinear dynamics in a linear framework. The Koopman operator predicts the system dynamics via the lifted representation: $z_{k+1+j|k}=(1-\sigma_{k})(A^{P}z_{k}+B^{P}u_{k}^{P})+\sigma_{k}(A^{D}z_{k}+B^{D}u_{k}^{D})$, where $\sigma_{k}$ distinguishes stance ($\sigma_{k}=0$) and swing ($\sigma_{k}=1$) phases. The Koopman MPC optimizes FES inputs to minimize a task-specific performance measure while adhering to state and control constraints, enabling real-time, phase-specific gait assistance. This framework effectively coordinates plantarflexion and dorsiflexion to support natural walking patterns.

The participants walked on a treadmill with FES applied on on the TA and GAS muscles during the swing and stance phases, respectively. The walking setup is illustrated in Fig. (3). The FES electrodes were placed on the fibular head and the lateral malleolus of the TA muscle. For plantarflexion, the negative electrode is placed on the head of GAS muscle and the positive electrode is placed above the Achilles tendon. The DDMPC algorithm described in (22) computed FES inputs to TA and GAS muscles. The switching between them was implemented with ground reaction force based gait phase detection indicator function, $\sigma_{k}$, to trigger stance and swing optimal stimulation for plantarflexion and dorsiflexion. The primary objective of these task was to avoid any foot drag and achieve adequate foot clearance (pitch, $20deg>x_{1}>-20deg$.) for each gait cycle during the entire trial.

The real-time implementation was implemented in MATLAB/Simulink (R2019b, MathWorks, MA, USA) and executed on a Speedgoat target machine (Speedgoat Inc., Liebefeld, Switzerland). The Koopman Model Predictive Control (MPC) was implemented using the Gradient-based Receding Horizon Model Predictive Control (GRAMPC) solver [26]. The GRAMPC algorithm used a prediction horizon of 0.1 seconds and a sampling rate of 200 Hz. The solver uses a gradient-based optimization approach, dynamically switching between controlling the tibialis anterior (TA) and GAS muscles based on a GRF-based gait phase detection indicator function.

The experiments were divided into 8 sessions where the first 2 sessions were used to generate Koopman operator characteristics. For implementing Koopman MPC, in each session we conducted 4 trials each at speeds 0.1m∕s, 0.2m∕s, and 0.3m∕s, that is 12 trials in total per session. Each trial was conducted with rest intervals of 5-7 minutes to recover from muscle fatigue. In total, 4 trials each at 3 different speeds across 3 sessions were conducted for each subject, that is, 36 trials in total.

**Fig. 4.**
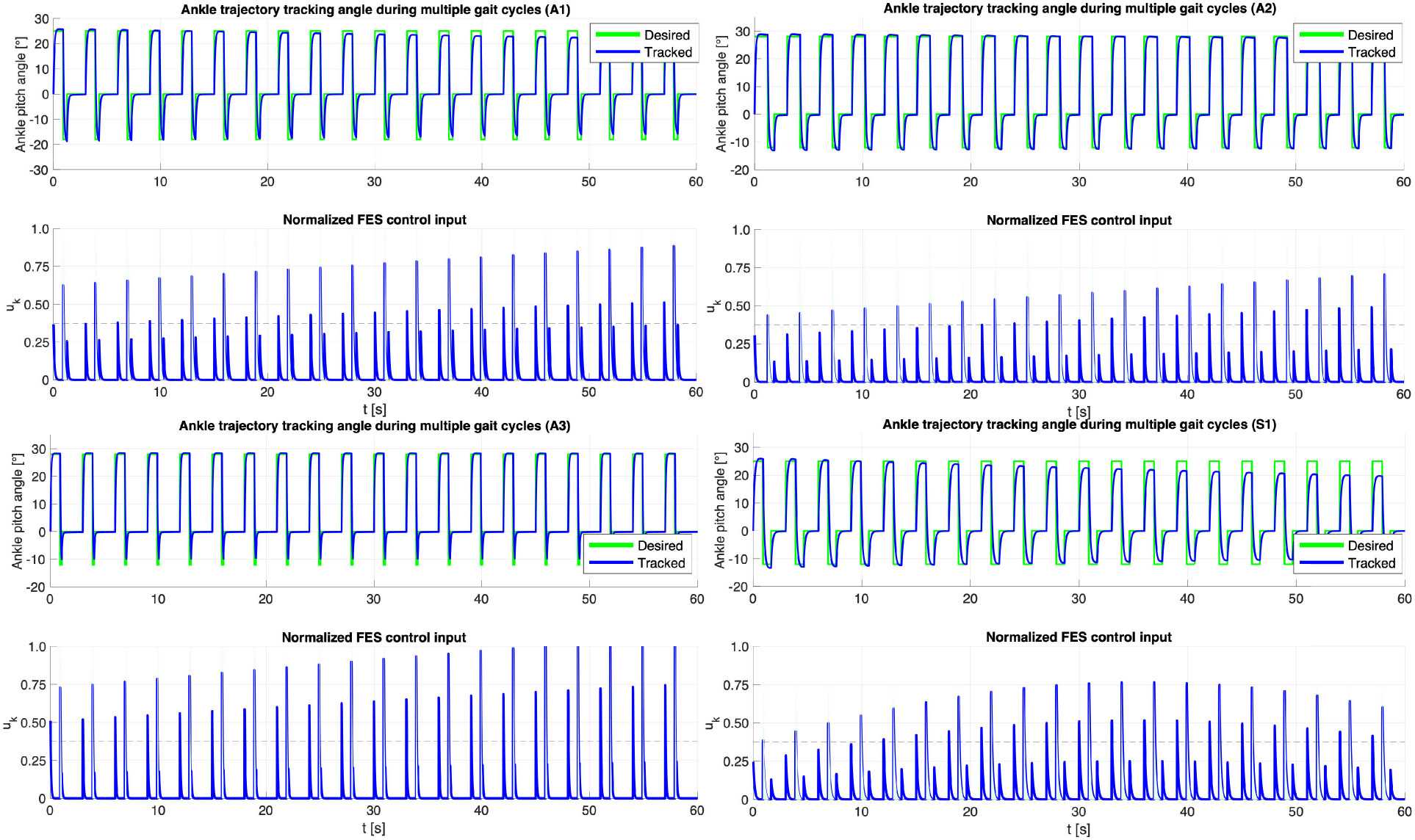
Trajectory tracking of ankle motion using DDMPC FES for subjects A1, A2, A3, and S1 (clockwise). The figure illustrates ankle motion trajectory tracking performance when the muscles are fully rested. The tracking achieved in this condition demonstrates a Root Mean Square Error (RMSE) of 1.625°, highlighting the system’s effectiveness in accurate control under optimal muscle conditions. Note, only absolute values of FES stimulation, u, are provided for both phases.

For each speed the first trial showed the best tracking results. The mean trajectory tracking plots for both plantarflexion and dorsiflexion for first trials across all speeds and sessions are presented in Fig. (4) and the RMSE metrics are presented in (II). The treadmill walking speeds in the current study were selected as 0.1m∕s, 0.2m∕s, and 0.3m∕s, due to the targeted clinical population with little to no volition in their affected leg. Successive trials across all sessions and speeds showed a drop in trajectory tracking due to muscle fatigue.

**Fig. 6.**
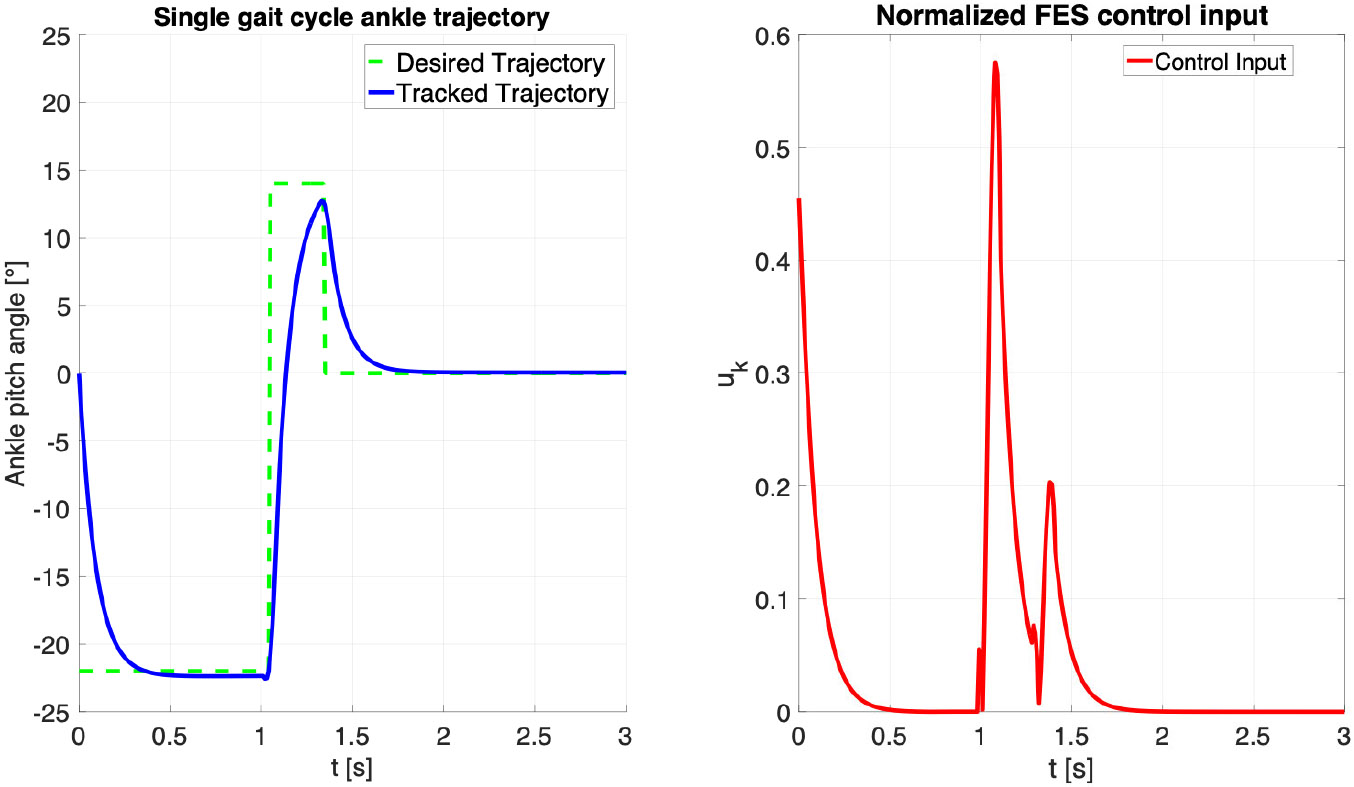
Comparison of the desired and tracked ankle pitch angle trajectories during a single gait cycle, achieved using a Koopman-based Model Predictive Control (MPC) framework.

**Fig. 7.**
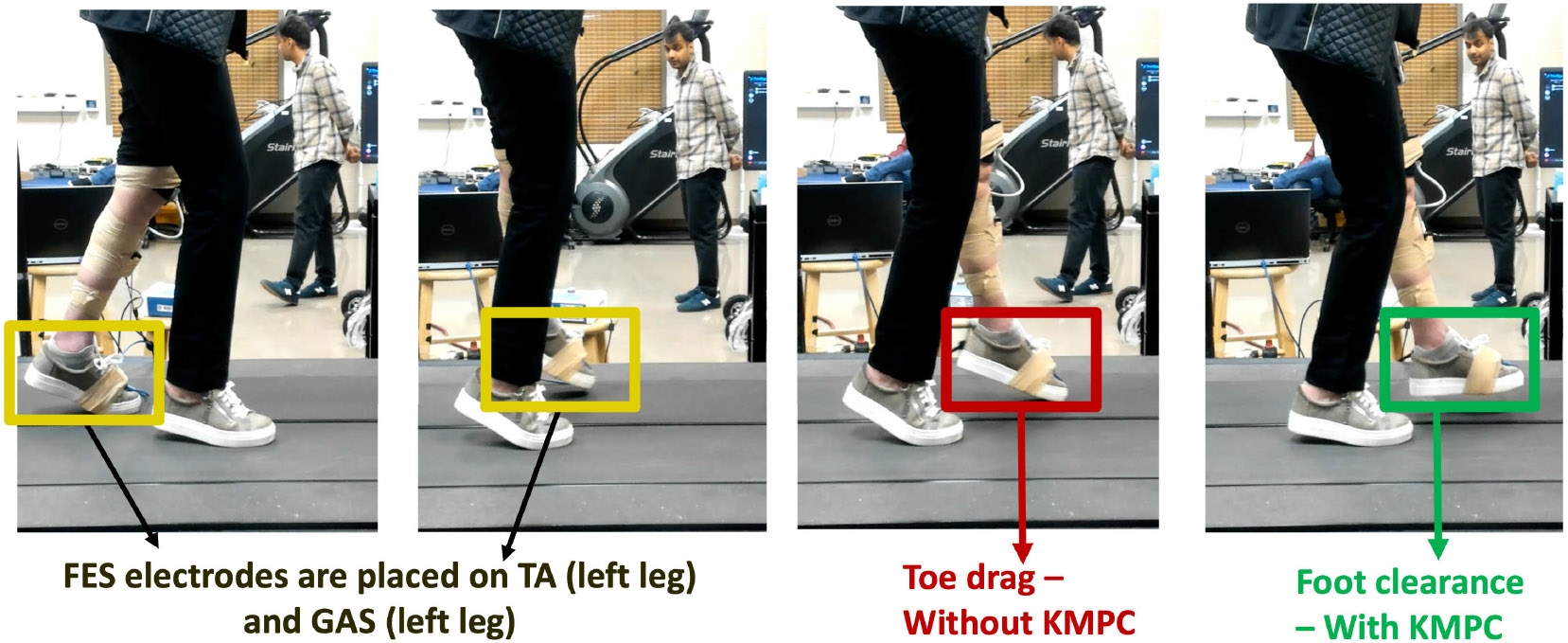
Comparison of gait performance for Subject S1 before and after using the FES-driven gait assist controller. A: Foot/toe drag observed prior to FES application, where the subject was unable to sustain walking on a treadmill at the lowest speed of 0.1 ms^−1^. B: No foot/toe drag observed after applying the FES-driven controller, enabling sustained walking at treadmill speeds of 0.1, 0.2, 0.3 ms^−1^. Annotations highlight the transition between pre-FES and post-FES conditions and the effectiveness of the proposed controller in supporting walking performance.

Trajectory tracking showed consistent ankle plantarflexion and dorsiflexion response actuated by FES using Koopman MPC. We observe that FES input saturated only for participant A3 but maintained good trajectory tracking. FES input for TA muscle always remained within the prescribed limits, which is an improvement to our past results presented in [17] and shows the benefit of using gait-specific MPC controller to design FES input ankle assistance during gait constrain the inputs. Effect of FES-driven gait assist in S1 is described in Fig. (7). Fig. (6) shows the trajectory tracking results for a single gait cycle. For experiments, the reference trajectory consists of set points representing the adequate plantarflexion and dorsiflexion angles for the stance, swing, and rest phases of the gait cycle (green dashed line). The tracked trajectory (blue solid line) demonstrates the controller’s ability to accurately follow these set points. Subplot (*left*) illustrates the trajectory tracking performance, while subplot (*right*) shows the corresponding control input (red solid line) applied to achieve the tracking.

*Remark 2:* (Reference Trajectory for Slow Speed Walking) The desired trajectories used in the experiments do not strictly satisfy the Lipschitz continuity assumption on velocity, but the slow gait speed ensures that the transition between phases remains physically realizable. Moreover, the ability of the Koopman-MPC framework to successfully track these trajectories despite their high-velocity transitions demonstrates the controller’s robustness in handling such conditions. Walking at very slow speeds (0.1 to 0.2m∕s) is common for rehabilitation applications, hence the transition between the stance and swing phases is assumed to be smooth. In the experiments, the reference trajectory is defined based on comfortable plantarflexion and dorsiflexion angles, ensuring that the desired motion aligns with natural ankle movements.

## VI. Discussion

In this work, we used the KOT approach that can efficiently linearize the nonlinear dynamics of human ankle allowing for the application of a linear MPC strategy for both plantarflexion and dorsiflexion control. This linearization facilitates the formulation of the MPC problem as a real-time solvable quadratic program. This approach also offers a high degree of adaptability. By continuously incorporating new data, the model can dynamically adjust to changes in the patient’s gait, such as variations in walking speed. This makes the system highly personalized, as it can cater to the specific requirements and progress of each individual patient. This approach is particularly suited to the complex neuromuscular ankle motion dynamics as it accounts for human variability in muscle response due to FES stimulations, but doesn’t actually require the exact individual system parameters.

We hypothesize that incorporating volitional muscle activity should lead to optimal design of GAS and TA FES stimulation levels which mitigate muscle fatigue effects which is a future direction for this work. For S1, we observe that swing phase is consistently of longer duration as compared to non-disabled subjects. Moreover, the trajectory tracking performance deteriorated over time. This is as expected as there is no volition for S1 in their left ankle. We now intend to combine a closed-loop ultrasound informed muscle activity information, described in [10], in our data-driven optimal FES control framework to improve trajectory tracking for longer duration of walking and at higher speed.

### A. Comparison With Existing Controllers

The table IV highlights the performance of the proposed Koopman-MPC framework compared to ILC framework [6], Iterative Timing Control [8], and Adaptive Control [10] for FES-based gait assistance. Koopman-MPC achieves the lowest trajectory tracking error (RMSE: 2–3°). Unlike previous controllers, which primarily focus on the swing phase, Koopman-MPC provides assistance during both the stance and swing phases, offering more comprehensive gait support by facilitating both dorsiflexion for toe clearance and plantarflexion for push-off. Moreover, while traditional methods require cycle-by-cycle resetting, Koopman-MPC performs continuous real-time optimization, ensuring greater adaptability and stability. Additionally, Koopman-MPC allows for the potential integration of physiological sensors (e.g., sEMG, ultrasound) to enhance controller adaptability, whereas prior approaches do not address this aspect, losing muscle activity-based performance enhancement in FES design. Computational efficiency is maintained at 30 ms per cycle, making it comparable to adaptive controllers while offering better stimulation phase coverage and lower RMSE. Performance metric can be adapted to incorporate physiological sensor to account for muscle activity and potentially mitigating muscle fatigue effects.

### B. Limitations and Future Work

The Koopman operator framework provides a linear prediction model for nonlinear dynamical systems, enabling effective integration with Model Predictive Control (MPC). However, it faces certain challenges, such as the need for finite-dimensional approximations of an inherently infinite-dimensional operator. The appropriate selection of observables that accurately capture the system’s dynamics remains critical for achieving robust and precise predictions. Limitations also arise in handling muscle fatigue and real-time variability in neuromuscular behavior, particularly in dynamic and repetitive tasks like gait rehabilitation. Future research will aim to enhance the Koopman MPC framework by integrating real-time feedback from physiological sensors, such as surface electromyography (sEMG) and ultrasound, to account for muscle activation and fatigue dynamics. Developing adaptive Koopman operator update laws that incorporate this physiological data will improve the MPC controller’s robustness and adaptability to changing muscle conditions. Incorporating muscle fatigue models directly as observables or leveraging real-time sensor feedback will enable dynamic adjustments to stimulation strategies, mitigating fatigue effects during repetitive gait cycles.

Additionally, addressing stability challenges introduced by switched dynamics between stance and swing phases—especially at faster gait speeds—will require the development of phase-specific stability laws based on minimum dwell time based Lyapunov methods. Future directions also include extending the Koopman MPC framework for multi-joint control and exploring scalable solutions for higher degrees of freedom.

## VII. Conclusion

We developed a data-driven Model Predictive Control (MPC) framework to assist with achieving the normal range of ankle motion during gait. Our approach leverages Koopman Operator Theory (KOT) to transform the inherently complex and nonlinear dynamics of FES-actuated ankle motion into a linearized representation. This linearization enables the application of efficient linear control techniques to a highly nonlinear system. The linear prediction model derived through KOT allowed us to formulate the MPC problem as a quadratic program, significantly enhancing the real-time feasibility, precision, and adaptability of the FES-driven control system.

The effectiveness and stability of our approach were validated through experimental trials involving three participants without disabilities and one participant with multiple sclerosis (MS). The results demonstrated precise trajectory tracking assistance for the developed Koopman MPC controller. The developed KOT-based MPC framework can be used to deliver effective, real-time, and personalized assistance for individuals with gait-related impairments, including those caused by MS, stroke, and incomplete spinal cord injury (SCI).

## Figures and Tables

**Fig. 5.**
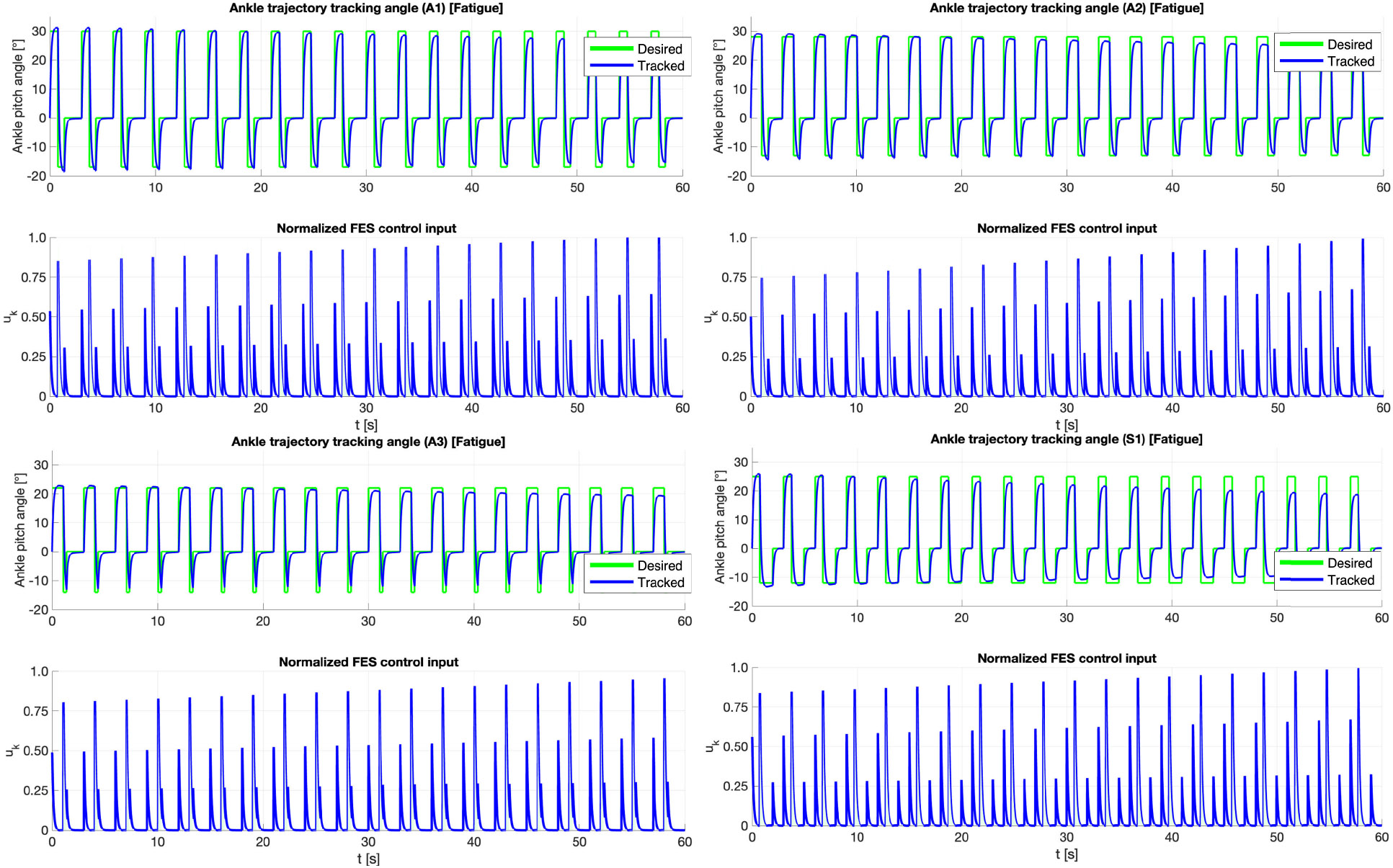
Ankle motion trajectory tracking results averaged over the final trial of each session for each participant A1, A2, A3, and S1 (*clockwise*).. The figure illustrates trajectory tracking performance after **3**–4 walking trials of 60 seconds each, reflecting the effects of muscle fatigue. The trajectory Root Mean Square Error (RMSE) is 3.1°, indicating the onset of fatigue-induced deviations in tracking accuracy.

**TABLE I:** Root Mean Square Errors ($\left\|{\tilde{x}}_{k}\right\|$) and Standard Deviation (σ) forselecting Appropriate Dictionary (Dict.). Custom, Original State, Trig. - Trigonometric Functions

PF	S1		A1		A2		A3	
Dict.	$\left\|{\tilde{x}}_{k}\right\|$	σ	$\left\|{\tilde{x}}_{k}\right\|$	σ	$\left\|{\tilde{x}}_{k}\right\|$	σ	$\left\|{\tilde{x}}_{k}\right\|$	σ
*Custom*	5.6	2.1	6.5	2.8	6.2	2.4	5.9	2.1
*State*	14.7	3.7	10.8	3.7	11.3	4.2	9.8	3.4
Trig.	2.3	0.8	1.6	0.9	1.8	0.7	2.0	1.1
DF	S1		A1		A2		A3	
Dict.	$\left\|{\tilde{x}}_{k}\right\|$	σ	$\left\|{\tilde{x}}_{k}\right\|$	σ	$\left\|{\tilde{x}}_{k}\right\|$	σ	$\left\|{\tilde{x}}_{k}\right\|$	σ
*Custom*	6.9	2.3	4.3	1.8	5.2	2.7	5.7	2.3
*State*	23.3	4.9	17.6	6.7	11.3	5.4	14.4	5.1
Trig.	3.4	1.8	2.1	0.8	2.8	0.7	2.5	1.1

**TABLE II:** Mean and SD Values of Anklejoint Trajectory Tracking for the Mean Gait Cycle for Subjects Forboth Plantarflexion (Left) and Dorsiflexion (Right) for Trial 1 at Speeds 0.1m∕s, 0.2m∕s, and 0.3m∕s Across All Sessions. 12 Trials Per Speed in Total. (Unit: °)

Speed	0.1 m/s		0.2 m/s		0.3 m/s		Speed	0.1 m/s		0.2 m/s		0.3 m/s	
Participant	Mean	SD	Mean	SD	Mean	SD	Participant	Mean	SD	Mean	SD	Mean	SD
S1	2.3	0.6	2.8	1.4	6.7	2.5	S1	1.4	0.4	2.2	0.8	4.7	1.3
A1	1.7	0.7	2.0	1.1	2.8	1.1	Al	0.8	0.2	1.5	0.3	2.5	0.8
A2	1.8	0.6	1.9	0.8	3.1	1.4	A2	0.9	0.3	1.9	0.3	1.9	1.1
A3	1.5	0.8	2.3	1.2	3.5	1.6	A3	1.1	0.5	1.3	0.4	2.1	0.7

**TABLE III:** Mean and SD Values Ofankle Joint Trajectory Tracking for the *Walking Task* forsuccessive Trials (Trials 2, 3 and 4) for Speeds 0.1m∕s, 0.2m∕s, and 0.3m∕s. 12 Trials Per Speed Per Session in Total. (Unit: °)

Speed	Trial 1 (0.1 m/s)		Trial 2(0.1)		Trial 3(0.1)m/s	
Participant	Mean	SD	Mean	SD	Mean	SD
S1	4.6	1.2	5.2	1.6	11.2	2.8
A1	2.3	1.3	2.8	1.6	2.6	1.5
A2	2.8	1.3	3.5	1.3	4.7	2.1
A3	3.2	1.7	4.2	2.4	4.5	2.6

**TABLE IV:** Performance ComparisonBetween Proposed Koopman-MPC and Past Controllers - ILC, PID, Adaptive- for FES-Based Ankle Joint Functionality Improvement

Controller	Koopman MPC	ILC [6]	Iterative Timing [8]	DSC [10]
RMSE (°)	2–8	4–8	1–7	5–9
Performance based optimization	Yes	No	No	No
Ankle Model	Koopman Linear	Linear	Linear	Nonlinear
Stimulation Phase	Stance and Swing	Swing	Swing	Swing
Real-Time Feasibility	Continuous real-time optimization	Requires resetting after each cycle	Requires timing resetting	Continuous trajectory tracking
Computational Cost (ms per cycle)	30ms	20ms	50ms	30ms
Patient Population	4 (1 MS; 3 AB)	6 (6 - Post Stroke)	10 (10 - Drop Foot)	5 (5 - AB)
